# Use of problem-based learning in orthopaedics education: a systematic review and meta-analysis of randomized controlled trials

**DOI:** 10.1186/s12909-024-05244-1

**Published:** 2024-03-08

**Authors:** Ting Li, Ruohong Song, Wenjie Zhong, Wenao Liao, Jiang Hu, Xilin Liu, Fei Wang

**Affiliations:** 1 Department of Orthopaedics, Sichuan Provincial People’s Hospital, University of Electronic Science and Technology of China, Chengdu, 610072 China; 2 Department of Cardiology, Sichuan Tianfu New District People’s Hospital, Chengdu, 610213 China; 3 Department of Postgraduate, Chengdu Sport University, Chengdu, 610041 China; 4 Department of Postgraduate, University of Electronics Science and Technology of China, Chengdu, 610054 China

**Keywords:** Meta-analysis, PBL, Traditional education, RCT

## Abstract

**Background:**

Currently, problem-based learning (PBL) has been widely used in many disciplines, but no systematic review has explored the advantages and disadvantages of PBL in orthopaedics education.

**Methods:**

We searched the PubMed, Cochrane Library, Embase, Web of Science, Scopus, Chongqing VIP Database (VIP), Chinese National Knowledge Infrastructure (CNKI), and Wanfang databases up to April 2023 to identify for relevant studies. Relevant studies were identified by using specific eligibility criteria, and data were extracted.

**Results:**

A total of 51 randomized controlled trials with 4268 patients were included. Compared with traditional education, PBL teaching yielded significantly higher knowledge scores (*SMD*=1.10, 95% CI: 0.78~1.41, *P*<0.00001), procedural skill scores and clinical skill scores than traditional teaching (*SMD*=2.07, 95% CI: 1.61~2.53, *P*<0.00001; *SMD*=1.20, 95% CI: 0.88~1.52, *P*<0.00001). Moreover, the total scores were higher in the PBL teaching group than in the traditional teaching group (*MD*=5.69, 95% CI: 5.11~6.26, *P*<0.00001). Students also expressed higher levels of interest and satisfaction in the PBL teaching group than in the traditional teaching group (*OR*=4.70, 95% CI: 3.20~6.93, *P*<0.00001; *OR*=5.43, 95% CI: 3.83~7.69, *P*<0.00001). However, there was less learning time and higher levels of learning pressure in the PBL teaching group (*OR*=0.12, 95% CI: 0.06~0.24, *P*<0.00001; *OR*=5.95, 95% CI: 3.16~11.23, *P*<0.00001).

**Conclusion:**

Current evidence indicates that PBL teaching can increase knowledge scores, procedural skill scores, and clinical skill scores. Students have higher levels of interest in teaching and higher levels of teaching satisfaction in the PBL group. However, students can feel higher levels of study pressure and experience less study time. The findings of the current study need to be further verified in multicentre, double-blind and large-sample RCTs.

**Supplementary Information:**

The online version contains supplementary material available at 10.1186/s12909-024-05244-1.

## Introduction

Problem-based learning (PBL) teaching was first proposed as an innovative form of medical education by McMaster University in the 1960s [1]. PBL is an education method that is student-centred and teacher-guided, and it uses practical problems as a learning context, thereby helping individuals to actively and innovatively acquire knowledge [2]. In contrast, traditional teaching methods are is teacher-centred because teachers use textbooks and multimedia presentations to impart knowledge to students, with the entire course being led by the teacher.

PBL teaching and traditional teaching methods have been widely used in many training programs under various circumstances, but traditional lecture-based teaching remains predominant in China [3, 4]. Clinical internships are crucial for medical students to develop their clinical reasoning and clinical skills. Orthopaedics is a discipline that requires a comprehensive knowledge system and rich clinical skills, with strict requirements for the mastery of human anatomy. It is currently difficult for traditional teaching methods to meet the learning needs of students. Therefore, some schools have incorporated PBL in orthopaedics education. PBL can help students better grasp knowledge and develop comprehensive problem-solving abilities [5]. It could also improve thinking and solving problems in real-life situations while enhancing cooperation and communication skills [6].

However, considering the different outcomes, such as knowledge and skill-related outcomes, PBL teaching was not superior to traditional teaching methods. Some studies have reported that PBL teaching is particularly difficult for time-constrained teachers and students because they are required to teach and learn with increasingly complex curricula [7–9]. Most Chinese students have not received PBL education since the beginning of primary school [10]. Hence, there is still some controversy regarding whether PBL teaching is appropriate in orthopaedics education. We aim to conduct a systematic review and meta-analysis of the current literature to explore outcomes related to the use of PBL in orthopaedics education.

## Materials and methods

### Study design

This systematic review and meta-analysis was performed in accordance with the Preferred Reporting Items for Systematic Reviews and Meta-Analyses (PRISMA) guidelines [11].

### Literature retrieval strategy

The following electronic databases were searched up to April 2023: PubMed, Embase, Cochrane library, Scopus, Web of Science, Chinese National Knowledge Infrastructure (CNKI), Chongqing VIP (VIP) and Wanfang. All RCTs comparing PBL teaching with traditional teaching were considered to be potentially eligible. The retrieval method adopted the combination of subject words and free words, and English retrieval words and Chinese versions include: (PBL OR [problem-based learning]) AND (Orthopaedics). Including articles were not any language restriction. In addition, the references of the included literature were reviewed to supplement the relevant studies.

### Inclusion and exclusion criteria

#### Inclusion criteria

The following inclusion criteria were developed based on the PICOS framework: 1) P: the target population was medical students, interns or resident doctors. 2) I: PBL teaching in the experimental group. 3) C: traditional teaching in the control group. 4) O: outcome: knowledge scores were used to assess how well the students the related theoretical knowledge; procedural skill scores, which were used to assess the operational skills, such as fracture reduction, fixation, and trauma management; clinical skill scores assessments, including medical history collection, physical examination, making diagnosis and treatment plan, were used to assess the ability of solving practical clinical problems; total scores, which included knowledge scores, procedural skill scores and clinical skill scores, were used to assess the overall abilities. What’s more, questionnaire surveys, were used to assess the different teaching methods, including teaching interest, teaching satisfaction, analysing and solving problem ability, learning time and learning pressure, independent learning ability, team assistance ability, communication ability, clinical reasoning ability, and so on. 5) S: RCTs were included.

#### Exclusion criteria

The exclusion criteria were as follows: 1) studies from which data could not be extracted; 2) duplicate reports; 3) students who received other forms of education; and 4) relevant outcome indices were not reported. 5) case report, letter, revision, technology note, commentaries, reviews, withdraw trails and meta-analysis.

### Data extraction

Two reviewers independently extracted the data from the included studies in accordance with the Cochrane Collaboration for Systematic Reviews guidelines. The two researchers independently read the full texts of potentially eligible studies that met the inclusion and exclusion criteria and extracted the following data: authors, year of publication, number of participants, intervention, comparison, study duration, and study design type.

### Quality assessment

The risk of bias in the included studies was evaluated using the Cochrane Handbook for Systematic Reviews tool to assess the risk of bias of the RCTs, and the Cochrane Collaboration’s tool for assessing the risk of bias is available online at http://hand-book.cochrane.org/ [12]. Bias assessments were independently carried out by two researchers. Any unresolved disagreements between reviewers were resolved through discussion or by evaluation by a third reviewer. The methodologic quality of each study was evaluated across on seven domains: random sequence generation, allocation sequence concealment, blinding of participants and personnel, blinding of the outcome assessment, incomplete outcome data, selective reporting, and other biases. Each item was rated as “low risk of bias”, “unclear risk of bias”, or “high risk of bias”.

### Statistical analysis

The RevMan 5.4 software package was used for this meta-analysis. Dichotomous outcomes are reported as odds ratios (ORs) with 95% confidence intervals (CIs), and continuous outcomes are reported as the mean differences (MDs) or standardized mean differences (SMDs) with 95% CIs. The chi-square test was used to assess heterogeneity. An I^2^ ≤50% indicated that there was little heterogeneity among the research results, and a fixed effects model was used. If *P*<0.05 and I^2^>50%, heterogeneity existed among studies, and a random effects model was used. We also performed a sensitivity analysis to identify the potential sources of heterogeneity. Publication bias was assessed with a funnel plot.

## Results

### Search results

**Fig. 1:**
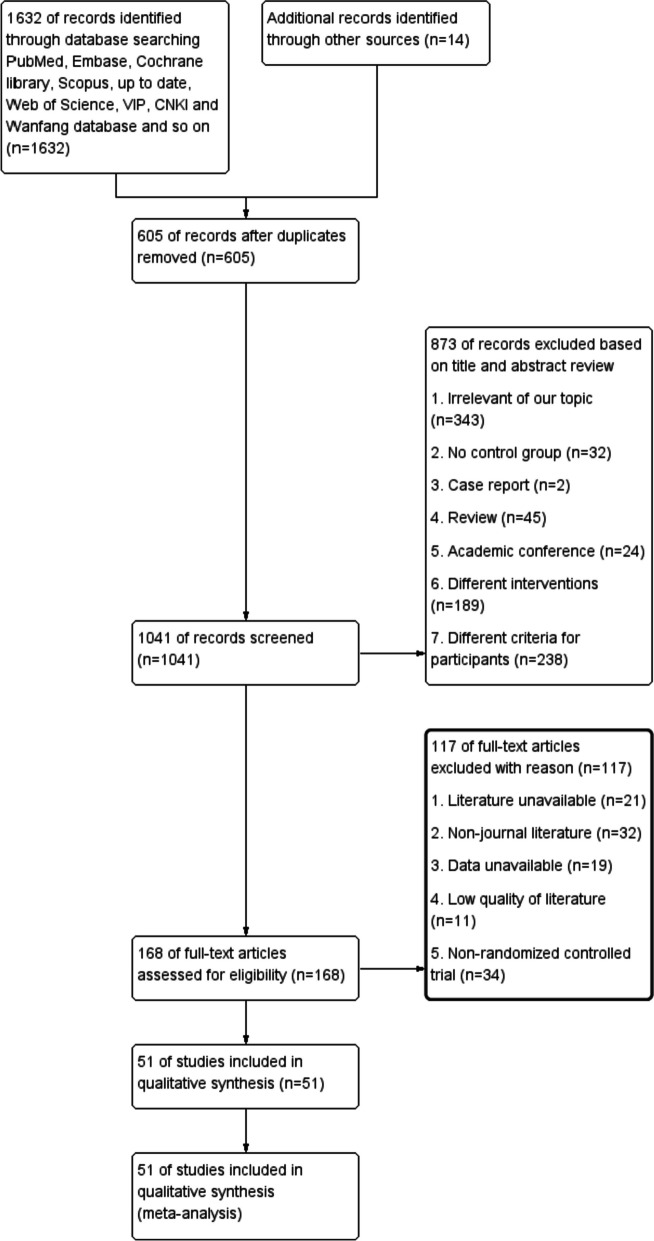
The flowchart of the study

The initial search yielded 1646 records, 605 of which were excluded due to duplication. After examination of the titles, abstracts and full texts of the articles, 51 potentially eligible studies met the inclusion criteria. After applying the inclusion criteria, 3 trials published in English and 48 trials published in Chinese were included in this meta-analysis. Figure 1 displays the selection algorithm and the numbers of included and excluded studies. All titles, abstracts, and texts were dually and independently reviewed by the authors based on the inclusion and exclusion criteria to minimize bias.

### Study characteristics

Fifty-one RCTs involving 4268 patients were included in this meta-analysis. All of the RCTs were published between 2006 and 2022, and they all assessed the effects of PBL compared with traditional teaching in orthopaedics education. The sample sizes ranged from 20 to 309. The majority of studies focused on undergraduates (*n*=23), with 21 studies for trainees, 2 for seven-year-old students, 2 for postgraduates, 2 for resident doctors and 1 for refresher doctors. Twenty-four studies were missing information on the age of the participants, and 31 studies were also missing duration data. The most frequent outcome was the theoretical knowledge score, which is used to assess how well students master the related theoretical knowledge. The scores on the clinical practice subscale evaluate the students’ clinical practice ability. The main basic characteristics of the included studies are shown in Table 1.

**Table 1:** Basic characteristics of the included literature

Name	Year	Study type	People	Age(I/C)	Number of persons (I/C)	Intervention group	Controlled group	Teaching subjects	Study duration
Zeng, JZ	2015	RCT	Five-year undergraduates	NA	30/29	PBL	LBL	Lumbocrural pain	2 months/2 months
Chen, HW	2017	RCT	Trainee	NA	15/15	PBL	LBL	Basic theory	NA
Chen, HF	2018	RCT	Trainee	23.4/23.4	104/92	PBL	LBL	Basic theory	NA
Chen, W	2019	RCT	Five-year undergraduates	21/21	53/53	PBL	LBL	Orthopaedic imageology	3 months/3 months
Ding, XY	2021	RCT	Trainee	19.25/19.21	40/40	PBL	LBL	Arthroscopy training	NA
Du, R	2021	RCT	Five-year undergraduates	19.7/21	20/20	PBL	LBL	Bone tumor	NA
Duan, G	2015	RCT	Trainee	NA	20/21	PBL	LBL	Basic theory	12 months/12 months
Duan, XL	2014	RCT	Trainee	NA	40/40	PBL	LBL	Basic theory	NA
Duan, XY	2014	RCT	Five-year undergraduates	NA	32/32	PBL	LBL	Basic theory	NA
Feng, ML	2019	RCT	Undergraduates	NA	20/19	PBL	LBL	Arthropathy	3 months/3 months
Gan, M	2020	RCT	Trainee	20.57/20.43	74/74	PBL	LBL	Basic theory	NA
Gao, ZR	2017	RCT	Postgraduate	NA	33/32	PBL	LBL	Basic theory	NA
Guo, WJ	2015	RCT	Five-year undergraduates	NA	30/30	PBL	LBL	Basic theory	4 months/4 months
He, CN	2021	RCT	Trainee	22.69/22.72	43/43	PBL	LBL	Orthopaedic imageology	12 months/12 months
Hu, Y	2018	RCT	Five-year undergraduates	NA	33/33	PBL	LBL	Lumbocrural pain	2 months/2 months
Li, JL	2018	RCT	Trainee	22-24/22-24	52/50	PBL	LBL	Orthopaedic failure cases	1 months/1 months
Li, LM	2006	RCT	Seven-year students	NA	30/30	PBL	LBL	Basic theory	5 months/5 months
Liu, PD	2019	RCT	Trainee	24.1/23.3	44/44	PBL	LBL	Basic theory	NA
Liu, W	2018	RCT	Trainee	22.98/23.06	40/40	PBL	LBL	Orthopaedic flap theory	NA
Liu, Y	2020	RCT	Five-year undergraduates	22.7/22.7	35/35	PBL	LBL	Basic theory	NA
Liu, YJ	2013	RCT	Five-year undergraduates	NA	10/10	PBL	LBL	Basic theory	NA
Liu, CL	2012	RCT	Trainee	NA	19/19	PBL	LBL	Basic theory	NA
Nie, H	2016	RCT	Trainee	NA	29/26	PBL	LBL	Basic theory	1 month/1 month
Wang, JY	2013	RCT	Trainee	NA	18/18	PBL	LBL	Basic theory	NA
Wang, KP	2019	RCT	Trainee	22.5/23.0	19/19	PBL	LBL	Basic theory	NA
Wang, MB	2018	RCT	Trainee	21.4/21.1	40/40	PBL	LBL	Trauma orthopaedics	NA
Wang, Q	2012	RCT	Five-year undergraduates	24/24	42/42	PBL	LBL	Basic theory	NA
Wang, XS	2015	RCT	Five-year undergraduates	24.5/24.5	30/30	PBL	LBL	Basic theory	NA
Wang, YF	2009	RCT	Undergraduates	NA	155/154	PBL	LBL	Basic theory	NA
Wei, M	2019	RCT	Undergraduates	NA	86/82	PBL	LBL	Traditional Chinese bone teaching	NA
Wu, K	2019	RCT	Trainee	22.34/22.57	32/32	PBL	LBL	Basic theory	NA
Wu, M	2013	RCT	Five-year undergraduates	22.2/22.2	36/36	PBL	LBL	Basic theory	12 months/12 months
Xi, YH	2014	RCT	Undergraduates	NA	73/73	PBL	LBL	Basic theory	NA
Xiao, WA	2017	RCT	Resident doctors	24.3/24.0	50/50	PBL	LBL	Basic theory	3 months/3 months
Yan, DL	2018	RCT	Undergraduates	NA	42/42	PBL	LBL	Basic theory	5 months/5 months
Yu, Y	2015	RCT	Undergraduates	NA	40/40	PBL	LBL	Basic theory	1 month/1 month
Yu, YF	2013	RCT	Five-year undergraduates	NA	35/35	PBL	LBL	Basic theory	NA
Yu, XH	2022	RCT	Undergraduates	22.04/21.92	25/25	PBL	LBL	Basic theory	2 months/2 months
Yu, C	2020	RCT	Trainee	22.50/21.89	30/30	PBL	LBL	Orthopaedic failure cases	2 weeks/2 weeks
Zhang, BX	2018	RCT	Trainee	21.5/21.6	40/40	PBL	LBL	Basic theory	NA
Zhang, H	2019	RCT	Five-year undergraduates	23.10/23.21	20/20	PBL	LBL	Arthroscopy training	NA
Zhang, LF	2020	RCT	Trainee	22.44/22.32	44/44	PBL	LBL	Arthropathy	NA
Zhang, L	2016	RCT	Trainee	22.1/22.1	56/56	PBL	LBL	Basic theory	NA
Zhang, W	2018	RCT	Undergraduates	24.1/23.5	40/40	PBL	LBL	Basic theory	NA
Zhang, YD	2017	RCT	Refresher doctors	27.2/26.8	45/45	PBL	LBL	Orthopaedic failure cases	2 months/2 months
Zhao, Z	2016	RCT	Five-year undergraduates	20-24/20-24	42/42	PBL	LBL	Orthopaedic failure cases	2 weeks/2 weeks
Zhou, LH	2020	RCT	Trainee	NA	21/21	PBL	LBL	Basic theory	1 month/1 month
Zhong, JW	2017	RCT	Seven-year students	NA	132/130	PBL	LBL	Basic theory	NA
Cong, L	2017	RCT	Resident doctors	NA	45/45	PBL	LBL	Basic theory	NA
Sun, MJ	2022	RCT	Five-year undergraduates	22.6/22.6	53/53	PBL	LBL	3D basic theory	NA
Zhao, X	2019	RCT	One-year post-graduate	NA	10/10	PBL	LBL	Orthopedic nurse	12 months/12 months

*RCT* Randomized controlled trial, *PBL* Problem-based learning, *LBL* Lecture⁃based learning, *NA* Not available

### The bias risk assessment results of the included studies

**Fig. 2:**
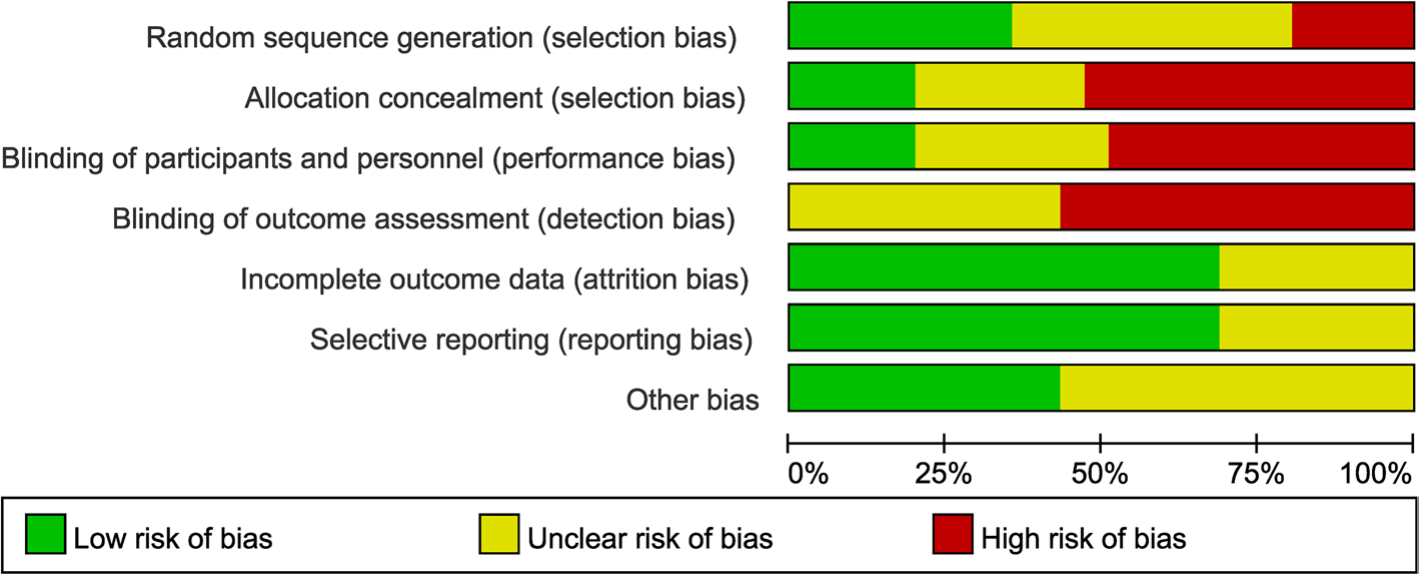
Results of quality assessment using the Cochrane risk tool

The risk of bias of RCTs was evaluated by the Cochrane tool. The authors showed the results of each quality item as percentages across studies. Ten studies were not randomized controlled trials (RCTs), 23 studies did not clearly describe the methods of random sequence generation, and 18 studies did apply a randomized controlled trial (RCT) design. The quality assessment of the included studies is shown in Fig. 2.

### Meta-analysis results

#### Knowledge scores

**Fig. 3:**
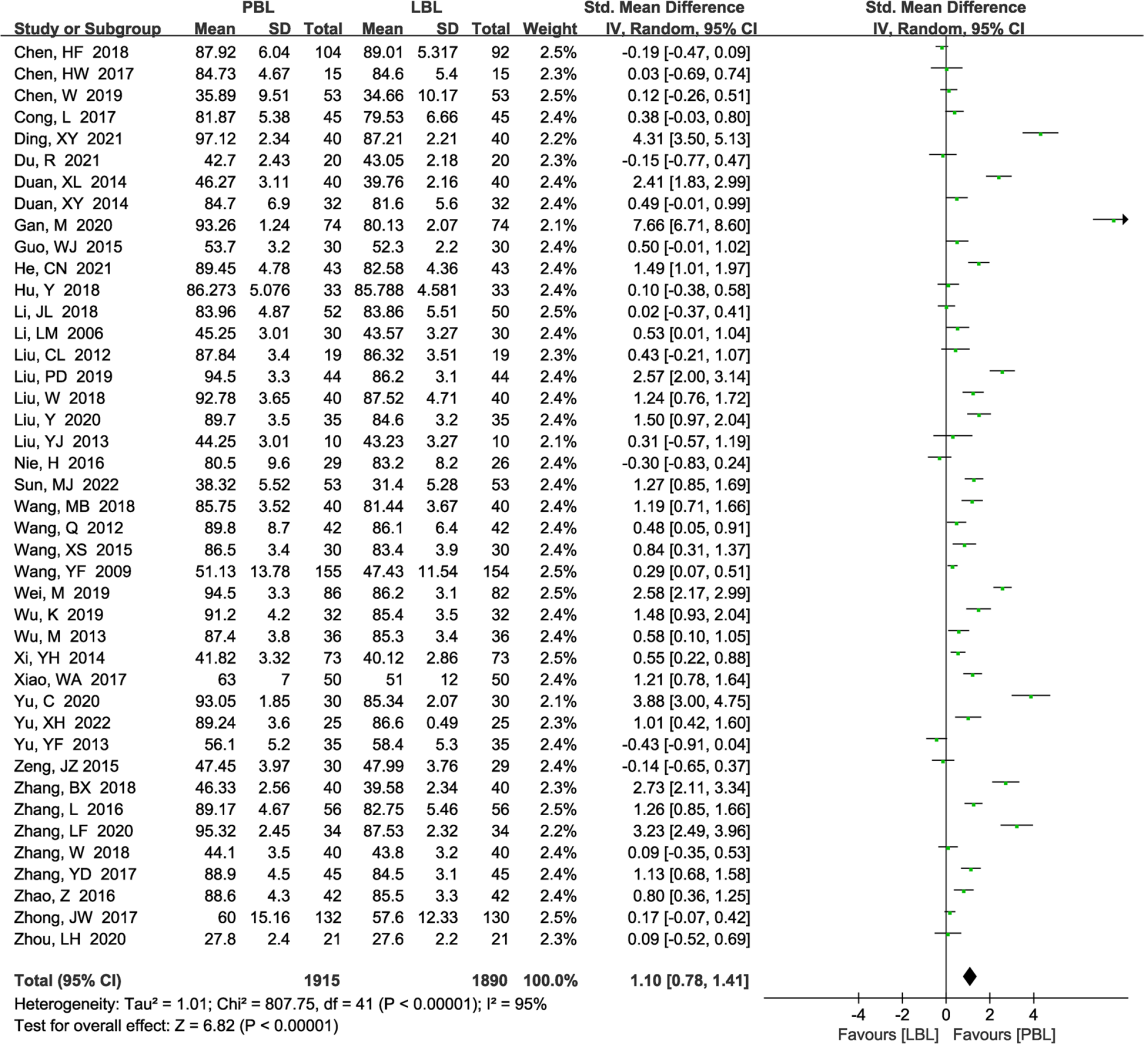
A forest plot showing the knowledge scores

A total of 42 [5, 13–53] studies (*N*=3805) reported knowledge scores. There was significant heterogeneity (*P*<0.00001, *I*^*2*^ =95%); therefore, a random effects model was used. We found that PBL teaching yielded higher knowledge scores than traditional teaching (*SMD*=1.10, 95% CI: 0.78~1.41, *P*<0.00001; Fig. 3). We performed sensitivity analysis to explore the potential sources of heterogeneity, but we failed to identify the sources.

#### Procedural skill scores

**Fig. 4:**
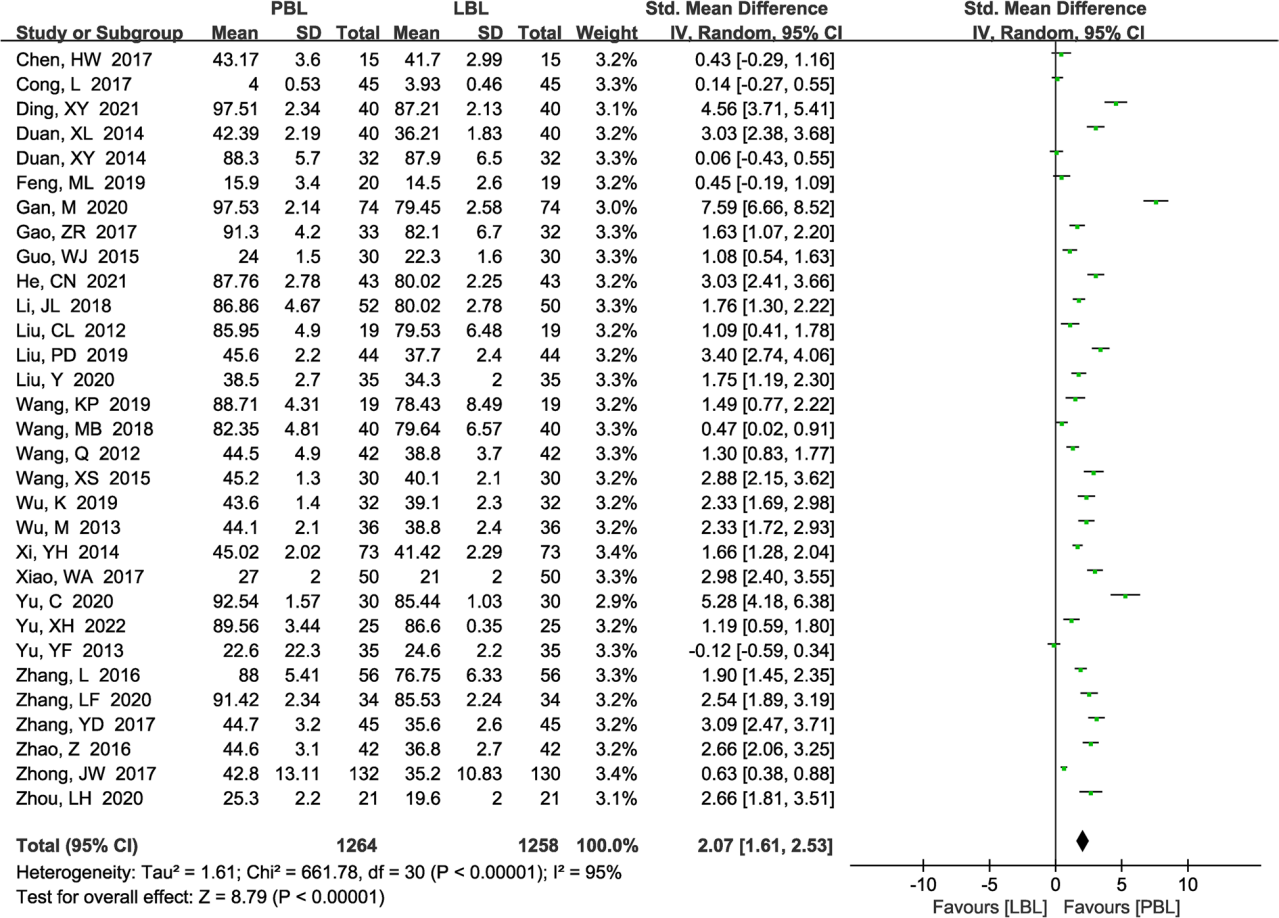
A forest plot showing the procedural skill scores

A total of 31 [14, 16, 17, 19–23, 25, 27, 28, 30, 33–35, 38–44, 47, 48, 50–56] studies (*n*=2522) reported procedural skill scores. There was significant heterogeneity among the studies; therefore, a random effects model was used (*P*<0.00001, *I*^*2*^ =95%). We found that PBL teaching yielded higher procedural skill scores than traditional teaching (*SMD*=2.07, 95% CI: 1.61~2.53, *P*<0.00001; Fig. 4). We also performed a sensitivity analysis to explore the potential sources of heterogeneity, but we failed to identify the sources.

#### Clinical skill scores

**Fig. 5:**
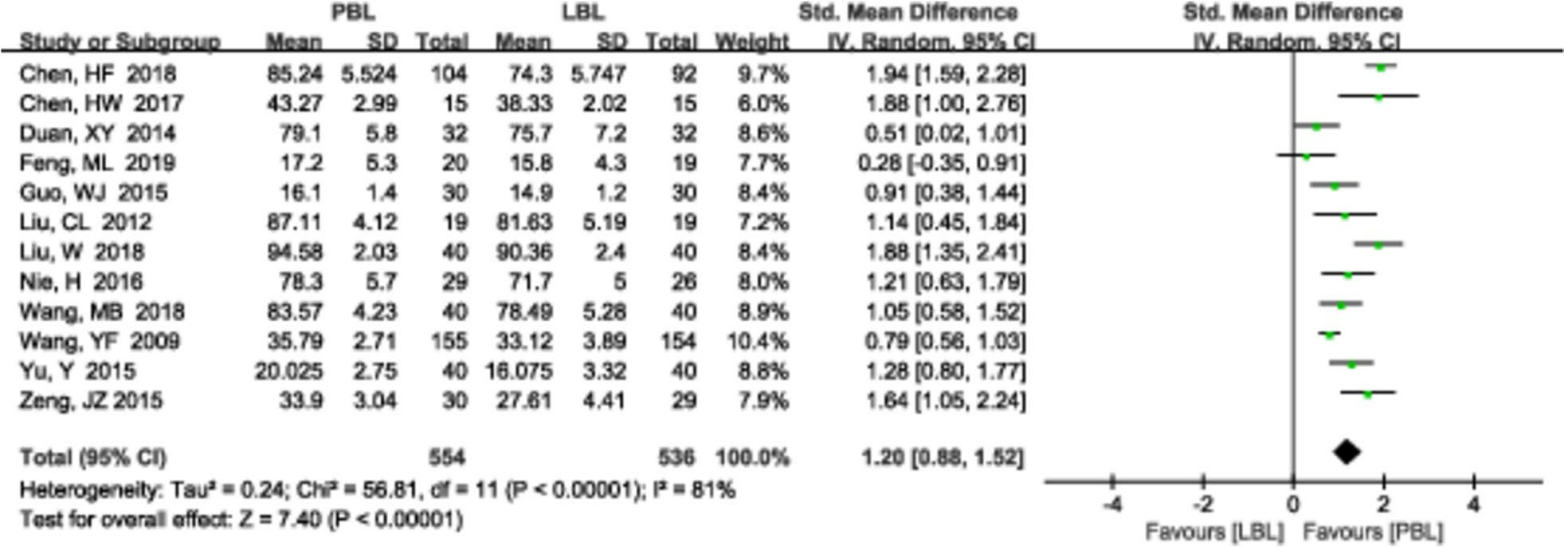
A forest plot showing the clinical skill scores

A total of 12 [13, 14, 17, 22, 27, 29, 32, 33, 36, 45, 54, 57] studies (*N*=1090) reported clinical skill scores. There was high heterogeneity (*P*<0.00001, *I*^*2*^ =81%); therefore, a random effects model was used. The meta-analysis results demonstrated that PBL teaching yielded higher clinical skill scores than traditional teaching (*SMD*=1.20, 95% CI: 0.88~1.52, *P*<0.00001; Fig. 5). We also performed a sensitivity analysis to explore the potential sources of heterogeneity, but we failed to identify the sources.

#### Total scores

**Fig. 6:**
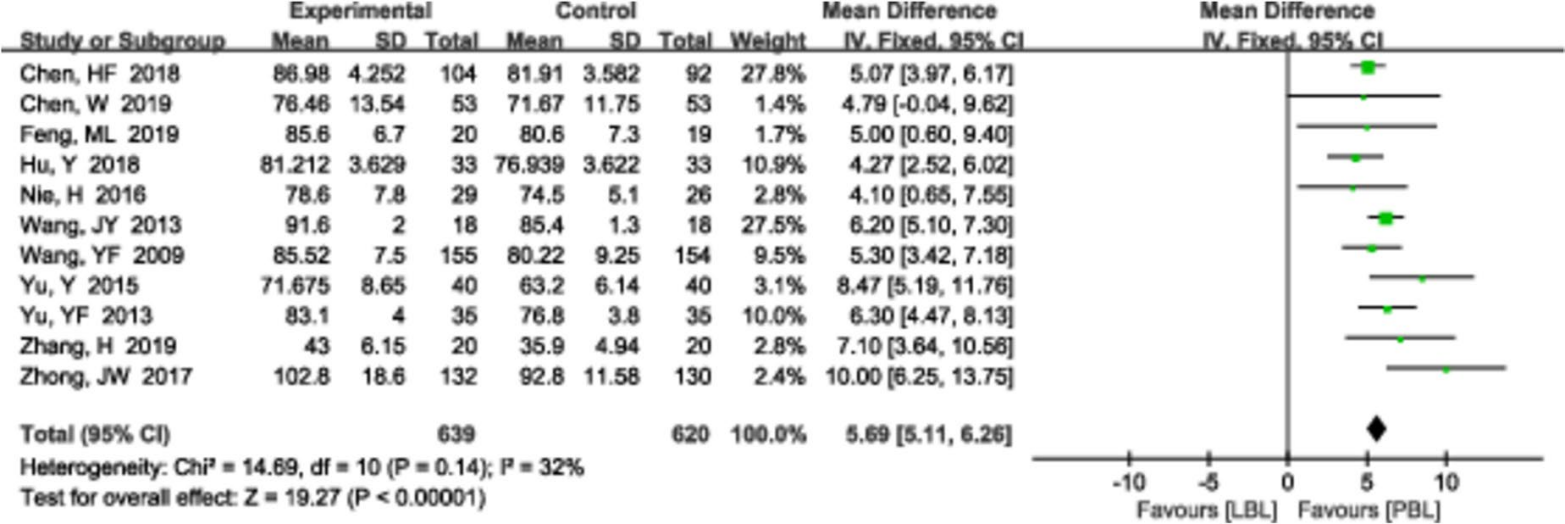
A forest plot showing the total score

A total of 11 [13, 15, 24, 32, 36, 44, 52, 54, 57–59] studies (*n*=1259) reported total scores. There was no significant heterogeneity (*P*=0.14, *I*^*2*^ =32%); therefore, a fixed effects model was used. The PBL group had higher total PBL scores than the traditional teaching group (*MD*=5.69, 95% CI: 5.11~6.26, *P*<0.00001; Fig. 6).

#### Teaching interest

**Fig. 7:**
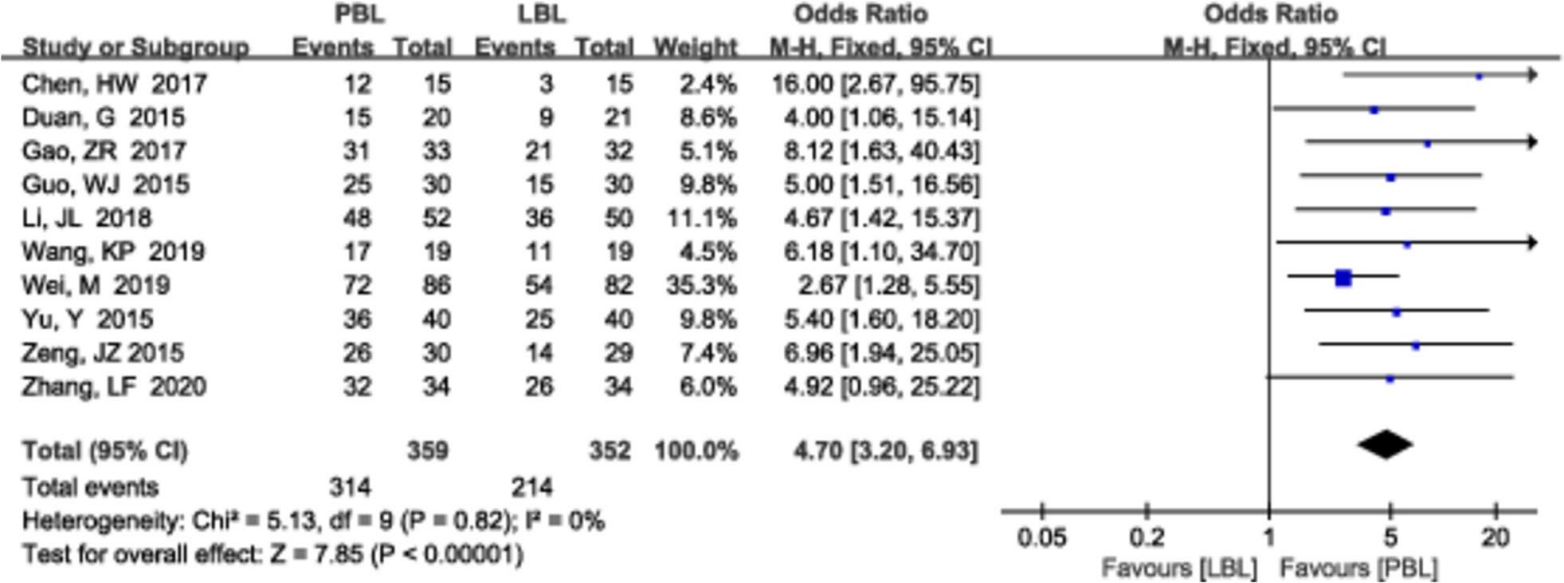
A forest plot showing the teaching interest

A total of 10 [14, 22, 25, 37, 45, 48, 55–57, 60] studies (*n*=711) reported interest in teaching. There was no heterogeneity (*P*=0.82, *I*^*2*^ =0%); therefore, a fixed effects model was used. This meta-analysis examined dichotomous outcomes and revealed that the PBL teaching group reported higher interest in teaching than the traditional teaching group (*OR*=4.70, 95% CI: 3.20~6.93, *P*<0.00001; Fig. 7).

#### Teaching satisfaction

**Fig. 8:**
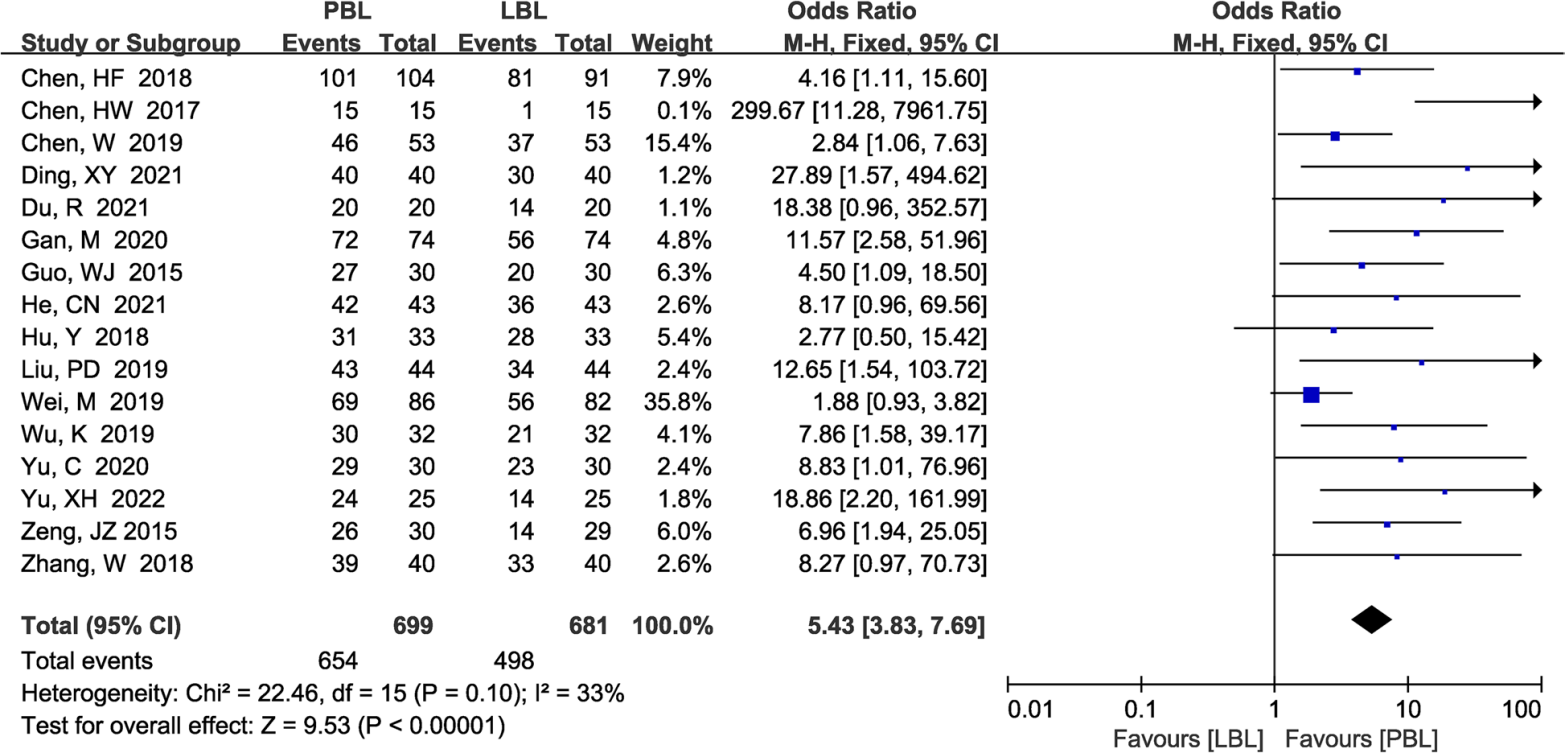
A forest plot showing the teaching satisfaction

A total of 16 [13–15, 17, 18, 21–24, 28, 37, 38, 42, 43, 45, 49] studies (*N*=1380) reported teaching satisfaction. There was no significant heterogeneity (*P*=0.10, *I*^*2*^ =33%); therefore, a fixed effects model was used. Our study demonstrated that PBL teaching yielded higher levels of teaching satisfaction than traditional teaching (*OR*=5.43, 95% CI: 3.83~7.69, *P*<0.00001; Fig. 8).

#### Analysing and solving problem ability

**Fig. 9:**
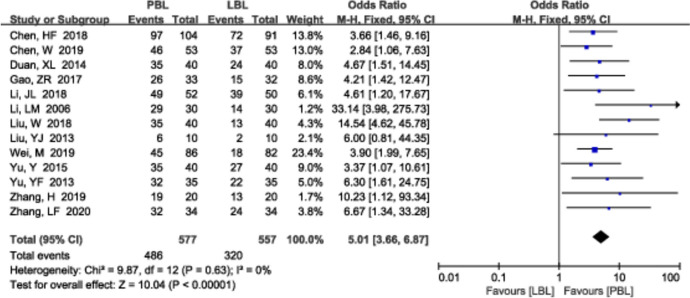
A forest plot showing the analysing and solving problem ability

A total of 13 [14, 15, 19, 25, 26, 29, 31, 37, 44, 48, 55, 57, 59] studies (*n*=1134) reported the ability to analyse and solve problems. There was no significant heterogeneity (*P*=0.63, *I*^*2*^ =0%); therefore, a fixed effects model was used. The ability to analyse and solve problems was significantly greater in the PBL teaching group than in the traditional teaching group (*OR*=5.01, 95% CI: 3.66~6.87, *P*<0.00001; Fig. 9).

#### Learning time and learning pressure

**Fig. 10:**
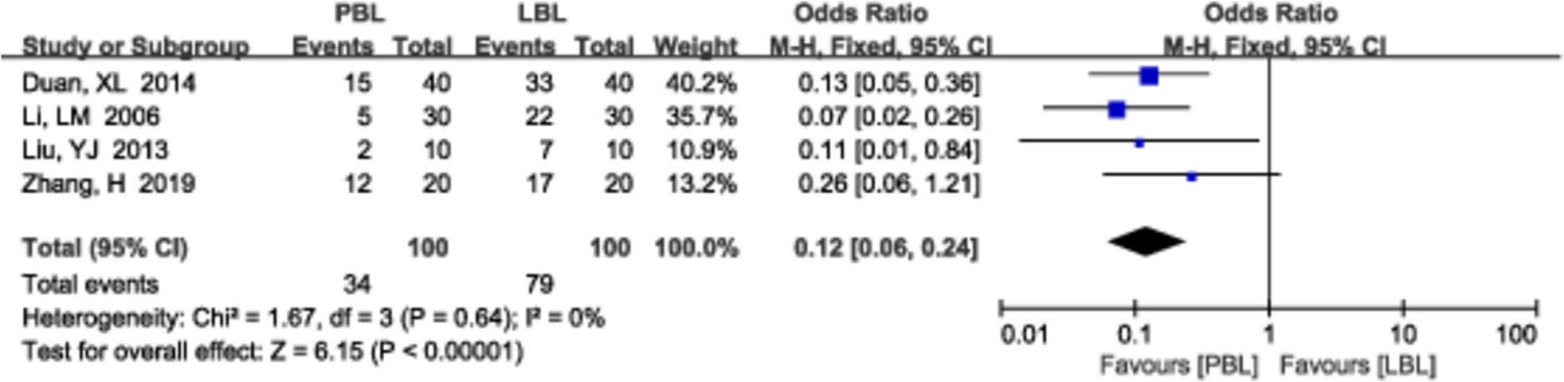
A forest plot showing the learning time

A total of 4 [19, 26, 31, 59] studies (*N*=200) reported learning time. There was no significant heterogeneity (*P*=0.64, *I*^*2*^ =0%); therefore, a fixed effects model was used. We found that there was less learning time in PBL teaching than in traditional teaching (*OR*=0.12, 95% CI: 0.06~0.024, *P*<0.00001; Fig. 10).

**Fig. 11:**
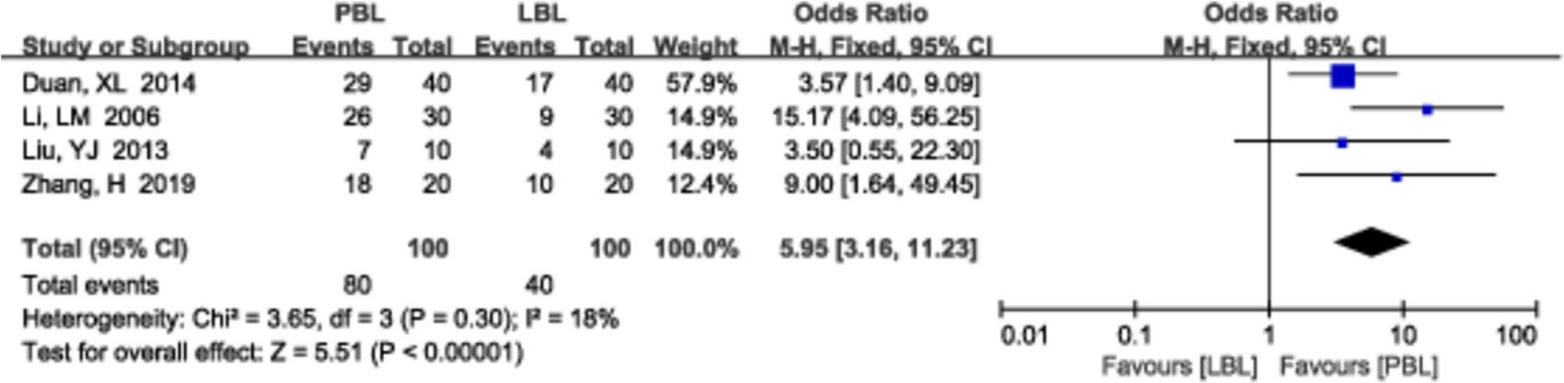
A forest plot showing the learning pressure

A total of 4 [19, 26, 31, 59] studies (*N*=200) also reported learning pressure. There was no significant heterogeneity (*P*=0.30, *I*^*2*^ =18%); therefore, a fixed effects model was used. We found that learning pressure was higher in the PBL teaching group than in the traditional teaching group (*OR*=5.95, 95% CI: 3.16~11.23, *P*<0.00001; Fig. 11).

#### Independent learning ability

**Fig. 12:**
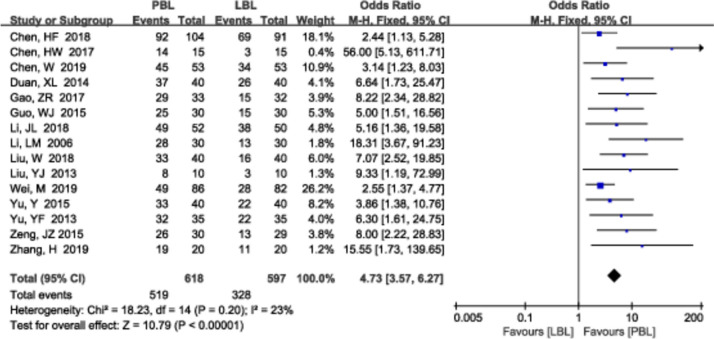
A forest plot showing the independent learning ability

A total of 15 [13–15, 19, 22, 25, 26, 29, 31, 37, 44, 45, 55, 57, 59] studies (*n*=1215) also reported independent learning ability. There was no significant heterogeneity (*P*=0.20, *I*^*2*^ =23%); therefore, a fixed effects model was used. We found that independent learning ability was higher in the PBL teaching group than in the traditional teaching group (*OR*=4.73, 95% CI: 3.57~6.27, *P*<0.00001; Fig. 12).

#### Team assistance ability

A total of 7 [14, 15, 19, 45, 55, 56, 59] studies (*N*=418) reported team assistance ability. There was no significant heterogeneity (*P*=0.45, *I*^*2*^ =0%); therefore, a fixed effects model was used. We found that team assistance ability was higher in the PBL teaching group than in the traditional teaching group (*OR*=4.16, 95% CI: 2.59~6.69, *P*<0.00001; see details in the Supplementary file: Figure S1).

#### Communication ability

A total of 8 [13, 14, 22, 28, 29, 45, 55, 56] studies (*N*=615) examined communication ability. There was no significant heterogeneity (*P*=0.58, *I*^*2*^ =0%); therefore, a fixed effects model was used. We found that communication ability was higher in the PBL teaching group than in the traditional teaching group (*OR*=4.24, 95% CI: 2.90~6.22, *P*<0.00001; see details in the Supplementary file: Figure S2).

#### Clinical reasoning ability

A total of 8 [14, 15, 18, 37, 45, 48, 57, 60] studies (*n*=592) reported clinical reasoning ability. There was no significant heterogeneity (*P*=0.27, *I*^*2*^ =21%). PBL teaching yielded superior clinical reasoning ability compared to traditional teaching (*OR*=3.75, 95% CI: 2.56~5.50, *P*<0.00001; see details in the Supplementary file: Figure S3).

#### Other outcomes

Our meta-analysis also reported other outcomes. We found that PBL teaching was not superior to traditional teaching in terms of the ability to grasp knowledge points and memory ability (Table 2: see details in the Supplementary file). PBL teaching was superior in terms of literature retrieval ability, expressive ability, and learning motivation (Table 2: see details in the Supplementary file). Moreover, we also found that PBL teaching was more effective than traditional teaching in terms of thinking and understanding abilities as well as in imaging reading ability (Table 2: see details in the Supplementary file).

**Table 2:** Other outcomes of the meta-analysis

Stratification	No. of studies	No. of patients	Pooled SMD/OR	95% CI of pooled SMD/OR	*P* value	Heterogeneity I^2^ (%)
Knowledge points	5	540	1.46	0.70 – 3.07	0.31	52
Literature retrieval ability	6	517	9.38	3.14 – 28.03	<0.0001	76
Expressive ability	10	669	6.05	4.22 – 8.68	<0.00001	25
Memory ability	3	231	1.46	0.81 – 2.63	0.21	0
Learning motivation	4	220	5.91	2.98 – 11.74	<0.00001	0
Think and understand ability	4	244	3.01	0.54 – 5.48	0.02	98
Imaging reading ability	5	404	1.10	0.28 – 1.91	0.009	93

*SMD* Standard Mean Difference, *OR* Odds Ratio, *CI* Confidence Interval

### Publication bias

**Fig. 13:**
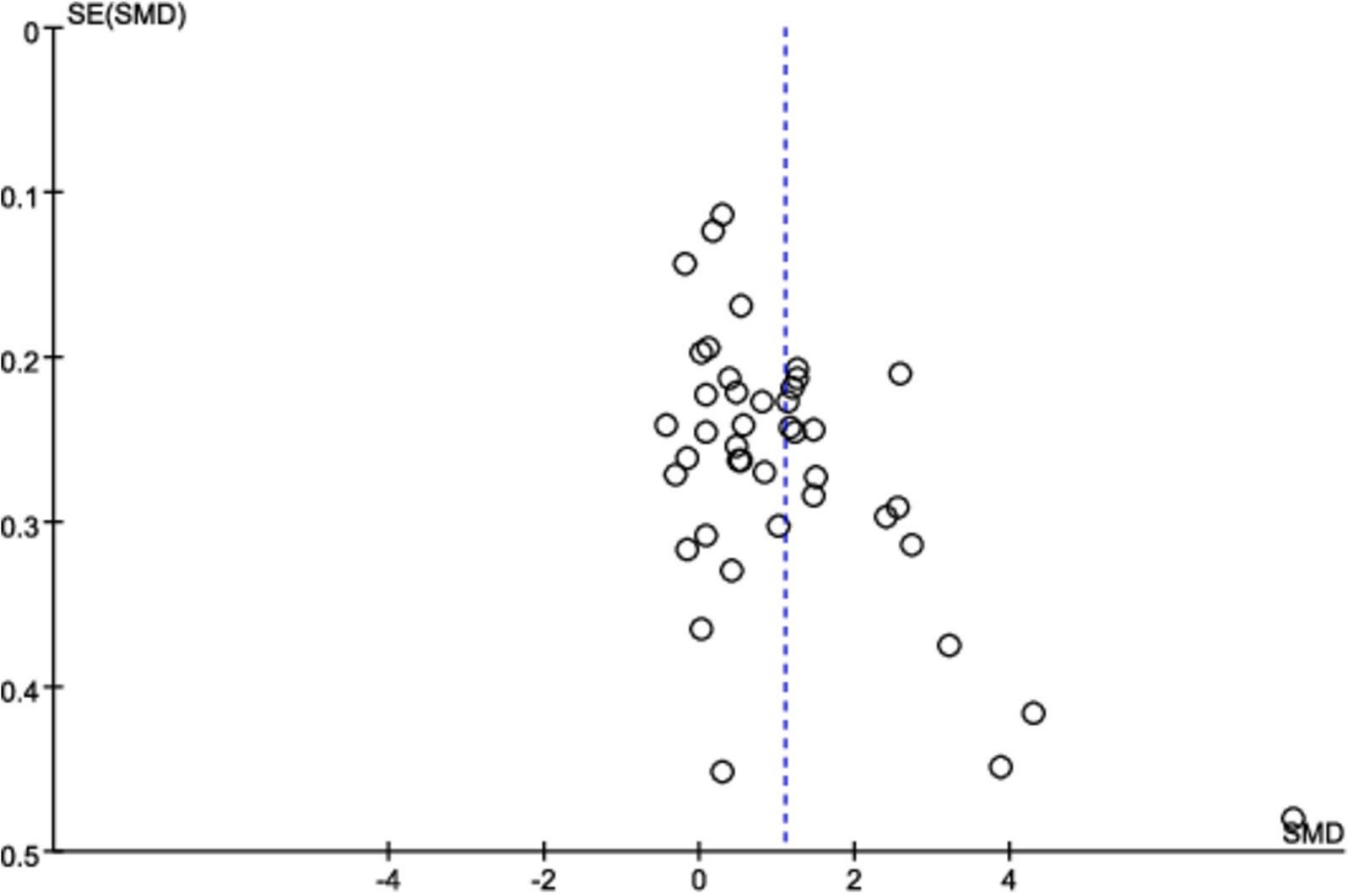
A funnel plot showing publication bias for knowledge scores

A funnel plot was used to evaluate the publication bias of the studies. For studies reporting knowledge scores, the funnel plot was symmetric (Fig. 13), indicating a lack of publication bias. Moreover, we detected publication bias in procedural skill scores (see details in the Supplementary file: Figure S4), clinical skill scores (see details in the Supplementary file: Figure S5), and total scores (see details in the Supplementary file: Figure S6).

## Discussion

The PBL teaching method is widely applied in China [61]. This method aims to improve academic performance, communication and collaboration skills, problem-solving abilities, and self-directed learning abilities [62, 63]. Orthopaedics is one of the most challenging areas because it covers a wide range of topics, including trauma, sports injuries, joints, and bone tumours. These concepts are not only abstract and complex but also closely related to disciplines such as anatomy, radiology, and biomechanics, making them difficult to comprehend and memorize. Hence, there is widespread adoption of PBL in orthopaedic education [64].

Traditional teaching is a type of passive education with a 'cramming' style, so students have a low level of initiative. As a result, students often have a rich theoretical foundation but lack clinical skill scores [65]. However, PBL teaching centres on professional issues to develop teaching plans and design learning content. PBL teaching can help students to enhance their ability to recall knowledge, thus leading to higher scores on theoretical tests than students who receive traditional teaching [66, 67]. Previous studies have shown that PBL teaching leads to higher exam scores than traditional teaching (*p*<0.05) [5]. Our findings also support this difference. However, when interpreting the results, it should be noted that there are many factors that can impact exam scores. Feeley et al. [68]. reported that many factors influence exam scores, including motivation, learning skills, and the length of study time, making it difficult to draw reliable conclusions about the impact of PBL versus traditional teaching on knowledge scores. However, numerous studies have shown that PBL teaching comprehensively enhances the overall abilities of students, including communication abilities, physical examination skills, the ability to conduct research, literature evaluation abilities and problem solving abilities [69, 70]. PBL teaching is guided by problems and clinical cases through case analysis, prompting students to discover related problems; thus, PBL teaching can stimulate students to explore causes and solve problems. This method, based on cases and assisted by teamwork, greatly increases the communication and collaboration skills of students, thereby allowing them to better adapt to the transition from student to doctor and laying a solid foundation for future clinical work [71]. Thus, PBL teaching can improve the communication and collaboration skills of students more than traditional teaching methods.

One of the most important parameters for evaluating a teaching method is student satisfaction. Our meta-analysis demonstrated that students express more interest and higher levels of satisfaction when receiving PBL education. PBL teaching can mobilize the subjective initiative of students, cultivate their learning ability, and increase their enthusiasm for learning. Norman et al. [72]. demonstrated that PBL teaching can enhance students’ learning interest and their ability to self-learn, as well as maintain these interests. Another study showed that professional knowledge and classroom satisfaction were superior in the PBL teaching group than in the traditional teaching group (*P*<0.05) [5]. Similarly, Sally et al. [73]. also showed that PBL teaching significantly improved the satisfaction of both students and teachers. This finding is consistent with our meta-analysis results. However, several key factors influence teaching satisfaction, including small group sizes and realistic case scenarios [74].

In research on PBL curricula in India and the United States, researchers have demonstrated that, compared to traditional teaching, PBL teaching not only has significant advantages in terms of teaching satisfaction but also achieves better results in terms of critical thinking, problem-solving skills, and communication skills [75, 76]. Our meta-analysis also reached the same conclusion. Moreover, PBL teaching utilizes heuristics, self-directed learning, and interactive discussions to explore answers to problems and cultivate independent thinking skills. Through communication among groups, oral expression skills and team spirit are cultivated. Furthermore, continuous reflection fosters creative thinking and logical reasoning skills, resulting in significant improvement in the ability to analyse and solve problems [77, 78]. However, Song et al. [79]. reported that PBL teaching was not superior to traditional teaching in terms of problem-solving skills. Our results contrast with this finding. Song et al. studied nurses, but our study included undergraduate medical students and physicians with higher education degrees. Therefore, the outcomes differed. Cultivating problem-solving skills is a complex process that requires time and involves comprehensive cognitive, attitudinal, and behavioural processes [80]. Therefore, the characteristics of students who receive PBL education may be a factor influencing the level of problem-solving skills.

Although PBL has many advantages, it also has several shortcomings. First, PBL teaching requires a high level of self-directed learning and collaboration skills. Students need to independently choose problems and solve problems based on their own interests. However, some students may lack the ability to independently learn and cooperate, which can lead to difficulties and frustration [81]. Second, although PBL focuses on personalized learning and practical applications, in some cases, students may not be able to participate in PBL due to various reasons, such as language barriers, physical disabilities, or learning disabilities [81, 82]. This can lead to a lack of inclusiveness in PBL teaching. More importantly, PBL requires more time and effort. Since PBL focuses on solving real-world problems, students need to spend more time and effort on independent thinking, self-directed learning, and collaborative problem solving [82, 83]. Our meta-analysis also revealed that learning time was lower and learning pressure was higher in PBL teaching (*OR*=0.12, 95% CI: 0.06~0.24, *P*<0.00001; *OR*=5.95, 95% CI: 3.16~11.23, *P*<0.00001). This may be due to the significant emphasis on self-study and group discussions in PBL teaching, which requires providing students with more learning space, time, and resources.

## Limitations

There are some limitations in our study. 1) First, all the studies we used in the meta-analysis were from China. In addition, some pooled results from the included studies were strongly subjective. Therefore, future studies should use larger samples from diverse locations. 2) We included only studies reported in English and Chinese, which may have led to language bias, and this might also have caused heterogeneity. 3) Half of the included studies considered did not provide detailed information on the frequency and duration of PBL interventions, and some studies also did not provide the average age of the participants. 4) Some studies exhibited significant heterogeneity. Although we performed a sensitivity analysis to explore the potential source of heterogeneity, there were still clinical outcomes for which heterogeneity was not found. Moreover, despite the inclusion of 51 RCTs, the risk of bias in the included studies increased due to the lack of information about randomization, allocation concealment, and blinding of outcome assessment in some studies. Hence, many large-sample RCTs are needed to decrease bias and to verify the clinical outcomes.

## Conclusion

This study showed that although students may experience less study time and higher levels of pressure in PBL teaching, PBL is beneficial. The knowledge scores and clinical skill scores were significantly higher than those of the traditional teaching group. Moreover, PBL teaching can enhance medical students' self-learning ability, clinical reasoning ability, problem-solving ability, communication and expression ability, and teamwork ability. Therefore, the PBL teaching model can significantly improve the quality of clinical teaching and is worth promoting and applying. However, these findings need to be further verified in multicentre, double-blind and large-sample RCTs.

### Supplementary Information

**Supplementary Material 1.** 

## Data Availability

All data generated or analysed during this study are included in this published article [and its supplementary information files].
