# Supplementary material for: Use of problem-based learning in orthopaedics education: a systematic review and meta-analysis of randomized controlled trials
Source: BMC Med Educ. 2024 Mar 8;24:253. doi: 10.1186/s12909-024-05244-1 (PMC10921736; doi:10.1186/s12909-024-05244-1)

**Figure S1：**A forest plot showing the team assistance ability


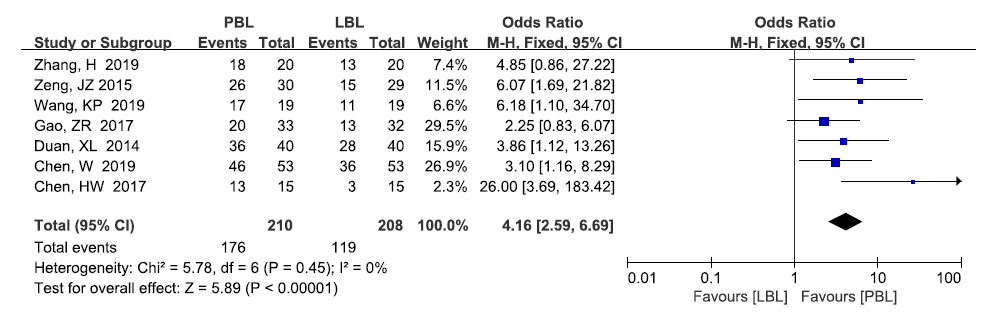


**Figure S2：**A forest plot showing the communication ability


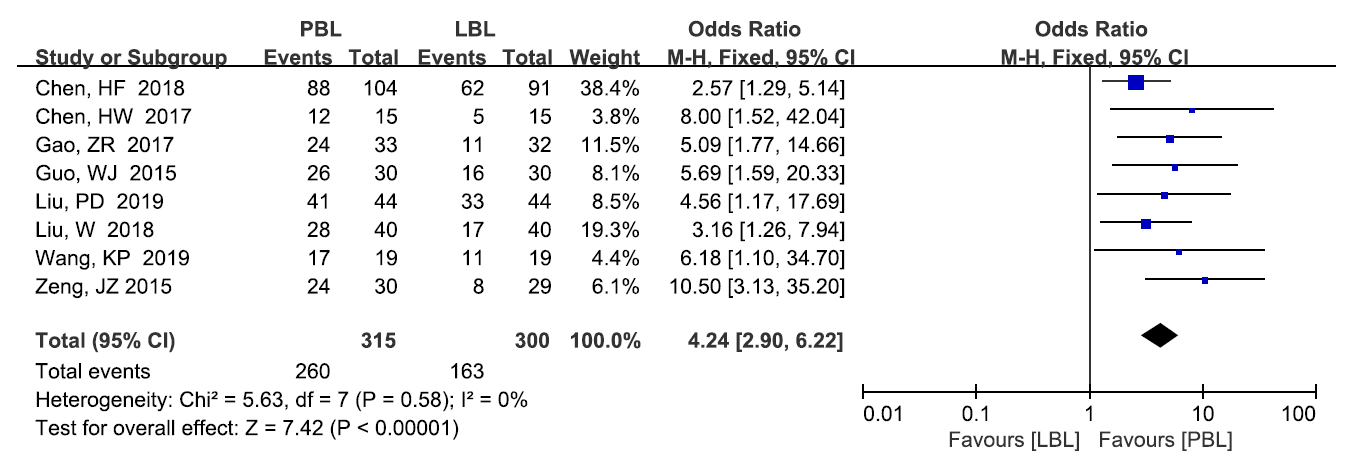


**Figure S3：**A forest plot showing the clinical reasoning ability


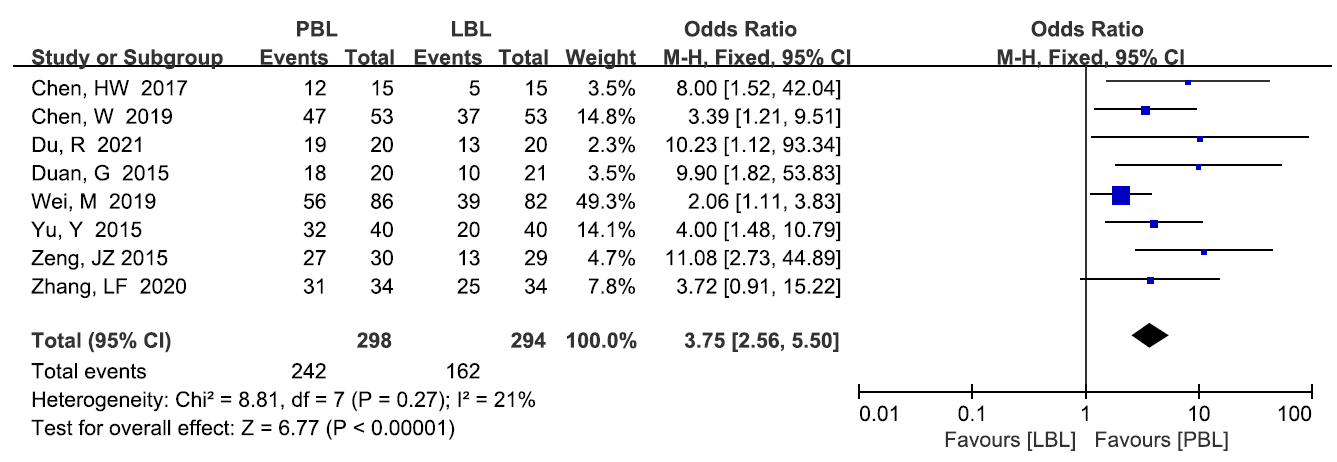


**Figure S4：**A funnel plot showing publication bias for procedural skill scores


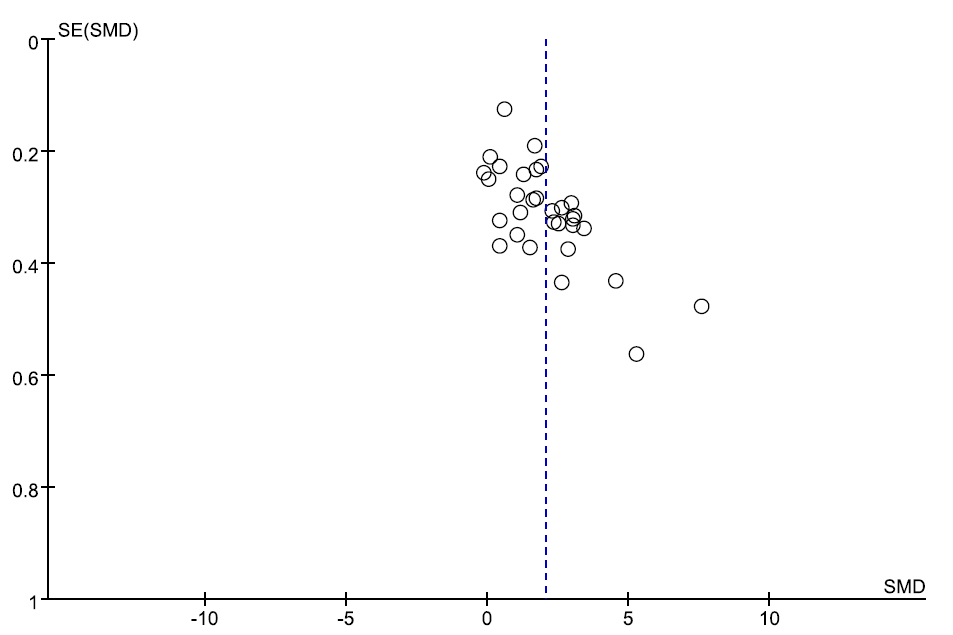


**Figure S5：**A funnel plot showing publication bias for clinical skill scores


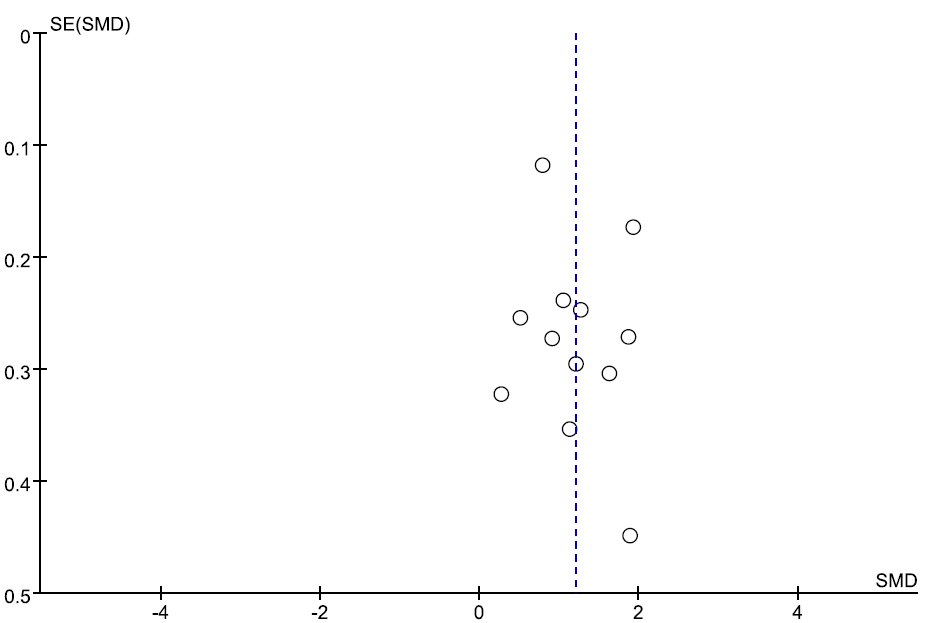


**Figure S6：**A funnel plot showing publication bias for total scores


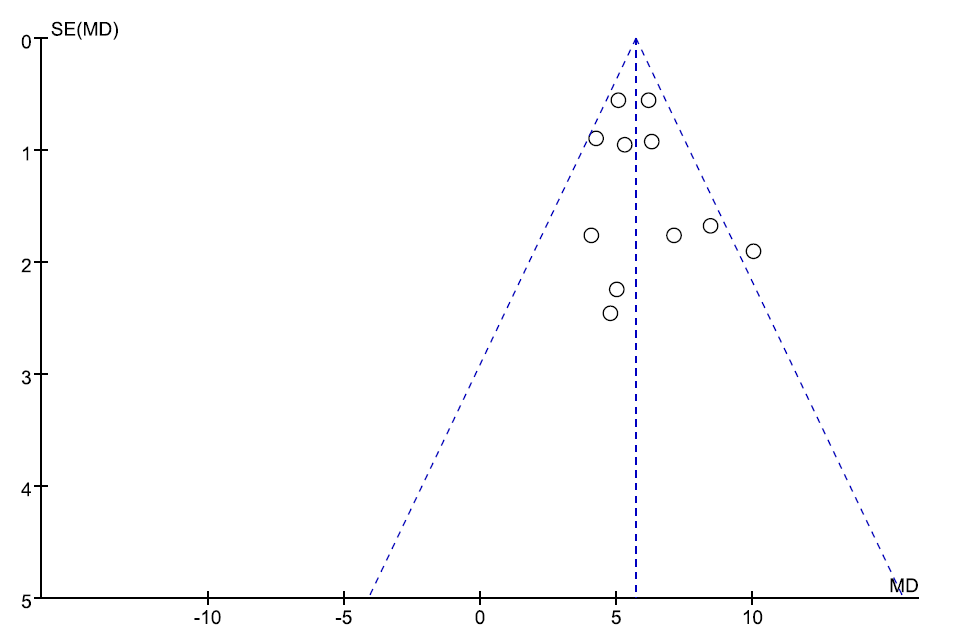

Supplement: Supplementary file 1 — Supplementary Material 1. [file 12909_2024_5244_MOESM1_ESM.docx]
